# Comparative Analysis of Microbial Diversity and Metabolic Profiles during the Spontaneous Fermentation of Jerusalem Artichoke (*Helianthus tuberosus* L.) Juice

**DOI:** 10.3390/plants13192782

**Published:** 2024-10-04

**Authors:** Tiandi Zhu, Zhongwang Li, Xinxing Liu, Chen Chen, Yuwen Mu

**Affiliations:** 1 Biotechnology Institute, Gansu Academy of Agricultural Sciences, Lanzhou 730070, China; ztd@gsagr.ac.cn (T.Z.); zwli-biotech@gsagr.cn (Z.L.); liuxx@gsagr.cn (X.L.); sjschench@gsagr.ac.cn (C.C.); 2 Agricultural Product Storage and Processing Research Institute, Gansu Academy of Agricultural Sciences, Lanzhou 730070, China

**Keywords:** Jerusalem artichoke, spontaneous fermentation, microbial diversity, secondary metabolites, flavor compounds

## Abstract

Jerusalem artichoke juice is valued for its nutritional content and health benefits. Spontaneous fermentation enhances its flavor, quality, and functional components through microbial metabolic activities. This study used high-throughput sequencing to analyze microbial community changes, and LC–MS and GC–MS to detect secondary metabolites and flavor compounds during fermentation. During natural fermentation, beneficial bacteria like *Lactobacillus* and *Pediococcus* increased, promoting lactic acid production and inhibiting harmful bacteria, while environmental bacteria decreased. Similarly, fungi shifted from environmental types like *Geosmithia* and *Alternaria* to fermentation-associated *Pichia* and *Penicillium*. A total of 1666 secondary metabolites were identified, with 595 upregulated and 497 downregulated. Key metabolic pathways included phenylpropanoid biosynthesis, with significant increases in phenylalanine, tryptophan, and related metabolites. Lipid and nucleotide metabolism also showed significant changes. Flavor compounds, including 134 identified alcohols, esters, acids, and ketones, mostly increased in content after fermentation. Notable increases were seen in Phenylethyl Alcohol, Ethyl Benzenepropanoate, 3-Methylbutyl Butanoate, Ethyl 4-Methylpentanoate, 5-Ethyl-3-Hydroxy-4-Methyl-2(5H)-Furanone, Ethyl Decanoate, Hexanoic Acid, and 1-Octanol. γ-aminobutyric acid (GABA) and other functional components enhanced the health value of the juice. This study provides insights into microbial and metabolic changes during fermentation, aiding in optimizing processes and improving the quality of fermented Jerusalem artichoke juice for functional food development.

## 1. Introduction

Jerusalem artichoke (*Helianthus tuberosus* L.) tubers are widely recognized for their high inulin content [1,2]. Inulin, which constitutes 10–25% of the fresh tuber weight, is a functional dietary fiber that acts as a prebiotic, promoting the growth of beneficial gut bacteria, such as bifidobacteria and lactobacilli, and thereby improving gut health [3,4]. Inulin significantly reduces blood glucose levels in diabetic patients and enhances insulin sensitivity [2,5]. Its hydrolysis product, fructose, does not cause a rapid increase in blood sugar levels, making it suitable for diabetic patients and those requiring glycemic control [6,7,8]. Additionally, inulin aids in the absorption of minerals like calcium and magnesium, promoting bone health, and playing a crucial role in preventing and treating osteoporosis [9,10,11,12,13].

Jerusalem artichoke tubers are also rich in antioxidants and anti-inflammatory compounds, which protect body cells by reducing oxidative stress and inhibiting inflammatory responses [14,15,16,17]. The high content of polyphenolic compounds and vitamins C and E in the tubers helps prevent cardiovascular diseases and cancer [15,18]. Furthermore, Jerusalem artichoke extracts can be used in fermented fruit and vegetable juices to boost probiotic content and functionality [15,18,19]. Jerusalem artichoke tubers improve the moisture retention and texture of fermented bread, increasing dietary fiber content and catering to health-conscious consumers [20,21,22,23]. The components of Jerusalem artichoke juice, rich in inulin and other fermentable sugars, serve as substrates for microbial metabolism, leading to the production of various primary and secondary metabolites [24].

Spontaneous fermentation is an effective food processing method that utilizes natural microbial communities from raw materials, equipment, and the environment [25,26]. This method preserves the original flavor of the ingredients while producing beneficial metabolites through microbial activity, thereby enhancing the nutritional and health value of the food [27,28]. Secondary metabolites play crucial roles in fermentation. Phenolic acids, flavonoids, and alkaloids exhibit antioxidant, antimicrobial, and anticancer properties. For example, flavonoids have strong antioxidant activity, protecting cells from oxidative stress [29,30]. Phenolic acids like chlorogenic acid and caffeic acid have anti-inflammatory and antimicrobial effects, enhancing food preservation and safety [31,32,33]. Additionally, alkaloids produced during fermentation, such as biotin and pyridoxine, improve the nutritional value of the food and enhance its health benefits [34,35]. Studies have shown that adding inulin to spontaneously fermented products like yogurt, kimchi, and fermented fruit and vegetable juices increases probiotic content and enhances health benefits. Inulin addition significantly improves the survival rate of probiotics and sensory properties in yogurt. During fermentation, inulin is metabolized by lactic acid bacteria, producing organic acids (e.g., lactic and acetic acids) and other metabolites that enhance the taste and nutritional value of yogurt [36,37,38,39,40].

The flavor of spontaneously fermented foods primarily arises from the various metabolites produced by microbes during fermentation. Through metabolic pathways, different microorganisms generate a variety of volatile compounds, such as alcohols, esters, aldehydes, ketones, and organic acids, creating complex and unique flavors and aromas. In the early stages of fermenting beer, sauerkraut, cocoa beans, carrot juice, and kimchi, common enterobacteria metabolize carbohydrates to produce various organic acids and alcohols, forming distinctive flavors [25,41,42]. Spontaneously fermented foods, free from artificial additives or industrial starter cultures, are more natural and pure, so are increasingly favored by consumers. Jerusalem artichoke tubers show broad application potential in spontaneously fermented foods, preserving the original flavor of the ingredients while generating various beneficial metabolites through microbial activity. With the growing demand for healthy and natural foods, the application prospects of Jerusalem artichoke tubers in spontaneously fermented foods are promising [43]. However, research on the microbial diversity and metabolic characteristics of spontaneously fermented Jerusalem artichoke juice remains limited.

In-depth studies on these characteristics are essential for understanding the dynamic changes in microbial communities and the mechanisms of metabolite production during the spontaneous fermentation of Jerusalem artichoke juice. This study aims to systematically analyze the structural and functional changes in microbial communities during the spontaneous fermentation of Jerusalem artichoke juice, elucidating the metabolic pathways of key microbes and their impacts on product flavor and nutritional value. This will help optimize fermentation processes, thus improving the quality and functionality of fermented Jerusalem artichoke products. Additionally, by investigating the secondary metabolites produced during spontaneous fermentation, including phenolic compounds, flavonoids, and alkaloids, we can better understand their health benefits and application potential in foods. Given the rich nutritional value and health benefits of Jerusalem artichoke tubers, this study will provide a scientific basis for developing high-value-added fermented products and promote the application and development of the Jerusalem artichoke in functional foods.

## 2. Materials and Methods

### 2.1. Sample Preparation

Fresh Jerusalem artichoke tubers of the cultivar “Lanyu No. 1” were sourced from the Yuzhong Experimental Station of Lanzhou University, Lanzhou, China (35°56′ N, 104°09′ E, 1750 m above sea level). The tubers were thoroughly washed with tap water, chopped into small pieces, and blended with distilled water at a ratio of 1:2 (*w*/*v*, 1 kg tuber to 2 L water) to obtain a homogenized slurry. The slurry was then filtered through an 80-mesh sieve to remove solid impurities. For each liter of juice, 50 g of white sugar was added. The juice was transferred into 2 L sterilized Erlenmeyer flasks, sealed with fermentation locks to allow CO_2_ to escape while preventing oxygen ingress, and incubated at 25 °C without agitation for 30 days to ensure sufficient fermentation time for the JASF samples. Unfermented juice samples served as control groups for comparative analysis (CK). Samples were collected, flash-frozen in liquid nitrogen, and stored at −80 °C for further analysis [44].

### 2.2. Determination of Microbial Diversity

To assess microbial diversity in the fermented juice, the samples were pretreated, and total microbial DNA was extracted. Samples (0.5 mL) were quickly thawed, placed in 2 mL centrifuge tubes with extraction lysis solution, and homogenized using a TissueLyser II (Qiagen, Hilden, Germany) at 60 Hz. Total DNA was extracted using the OMEGA Soil DNA Kit (M5635-02) (Omega Bio-Tek, Norcross, GA, USA), following the manufacturer’s protocol. The quality of the extracted DNA was assessed using a NanoDrop ND-2000 spectrophotometer (Thermo Fisher Scientific, Waltham, MA, USA) and 0.8% agarose gel electrophoresis.

The V3–V4 region of the bacterial 16S rRNA gene was amplified using primers 338F and 806R. For fungi, the internal transcribed spacer (ITS) region was amplified using primers ITS5 and ITS2. Each 25 μL PCR reaction contained 5 μL of 5× buffer, 0.25 μL of Fast Pfu DNA polymerase (5 U/μL), 2 μL of 2.5 mM dNTPs, 1 μL of each primer (10 μM), 1 μL of DNA template, and 14.75 μL of double-distilled H_2_O. Thermal cycling conditions for bacterial amplification were as follows: initial denaturation at 98 °C for 5 min; 25 cycles of denaturation at 98 °C for 30 s, annealing at 53 °C for 30 s, and extension at 72 °C for 45 s; with a final extension at 72 °C for 5 min. For fungal amplification, the annealing temperature was 55 °C, and the number of cycles was increased to 30 [45].

PCR products were purified using Vazyme VAHTS DNA Clean Beads (Vazyme, Nanjing, China) and quantified with a Quant-iT PicoGreen dsDNA assay kit (Invitrogen, Carlsbad, CA, USA). Equimolar amounts of purified amplicons were pooled, and paired-end 2 × 250 bp sequencing was performed on the Illumina NovaSeq platform at Shanghai Bioprofile Technology Co., Ltd., Shanghai, China.

### 2.3. Analysis of Secondary Metabolites

Samples were thawed and vortexed, and 100 μL of each sample was mixed with 100 μL of 70% methanol containing the internal standard, 2-chlorophenylalanine, at a concentration of 1 mg/L. The mixture was vortexed briefly and then incubated for 15 min at 4 °C, followed by centrifugation. The supernatant was filtered through a 0.22 μm membrane and stored for subsequent LC–MS/MS analysis. Samples were analyzed using an ACQUITY Premier HSS T3 column with a gradient of solvent A (0.1% formic acid in water) and solvent B (0.1% formic acid in acetonitrile) for the LC component, while the MS analysis was conducted in both positive and negative ion modes. The gradient conditions and parameters were optimized to ensure comprehensive metabolite detection. Mass spectrometry was conducted on an Agilent 6545A QTOF (Agilent, Santa Clara, CA, USA), with specific ion source parameters set for each mode.

### 2.4. Analysis of the Volatile Flavor Compounds Using HS–SPME–GC–MS

Each sample (1 mL) was mixed with a saturated NaCl solution and 20 μL of internal standard (10 μg/mL 3-Hexanone-2,2,4,4-d4) and extracted using HS–SPME for GC–MS analysis. GC–MS analysis was performed on an Agilent 8890-7000D system equipped with a DB-5MS column. Headspace extraction was conducted at 60 °C for 15 min using a 120 µm DVB/CWR/PDMS fiber, followed by desorption at 250 °C for 5 min. High-purity helium was used as the carrier gas at a flow rate of 1.2 mL/min. The temperature program started at 40 °C (held for 3.5 min), ramped to 100 °C at 10 °C/min, then to 180 °C at 7 °C/min, and finally to 280 °C at 25 °C/min, where it was held for 5 min. Mass spectrometry was conducted in electron impact ionization (EI) mode. Metabolites were identified and quantified using a custom database, with accuracy ensured through retention time and selective ion monitoring (SIM).

### 2.5. Statistical Analysis

Sequence data were processed using QIIME2 (version 2022.11) with taxonomic classification against the SILVA 132 database for bacteria and the UNITE 8.0 database for fungi. Alpha and beta diversity metrics were calculated and visualized using QIIME2 and R software (version 4.0.2), with differences in microbial communities assessed using PERMANOVA. Metabolomic data were processed with XCMS for peak detection, alignment, and retention time correction; metabolites were identified using public databases, such as HMDB and METLIN. Differential metabolites were analyzed using partial least squares discriminant analysis (PLS–DA) with SIMCA-P 14.0. Volatile compound data were expressed as mean ± standard deviation (SD), and statistical analyses were performed using IBM SPSS Statistics 23.0, with significance set at *p* < 0.05. All experiments included at least three biological replicates.

## 3. Results and Discussion

### 3.1. Changes in Microbial Diversity during Fermentation

#### 3.1.1. Bacterial Diversity

**Figure 1:**
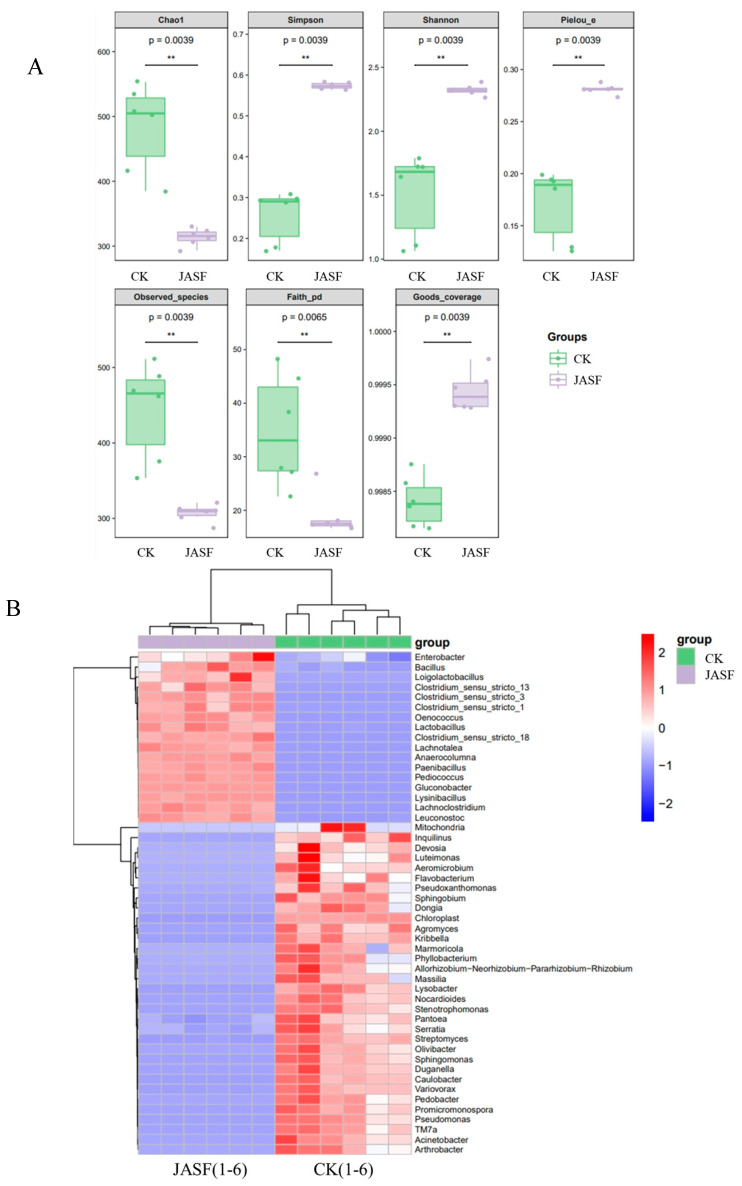

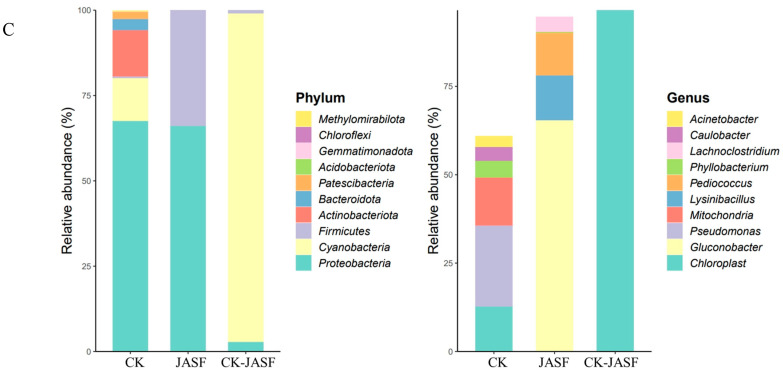
Composition and diversity of bacterial communities during spontaneous fermentation. α-diversity index analysis of bacteria (**A**). Heatmap of genus-level species composition for co-clustering (**B**). Species diversity and abundance of bacteria at the phylum and genus levels (**C**). Statistical significance is indicated by ** (*p* < 0.01).

A comprehensive analysis of the microbial communities in Jerusalem artichoke juice before (CK group) and after fermentation (JASF group) revealed significant shifts in microbial diversity (Figure 1A). The Chao1, observed species, and post-fermentation Faith_PD indices showed a marked reduction in diversity. Specifically, Chao1 decreased from approximately 483 to 314, observed species dropped from 443 to 307, and Faith_PD fell from 35 to 19. This suggests a dominance of certain microbial taxa during fermentation, resulting in decreased species richness. In contrast, the Simpson and Shannon indices, which reflect diversity and evenness, displayed an increase, with the Simpson index rising from 0.26 to 0.57 and the Shannon index from 1.5 to 2.3, indicating that, despite the reduction in species richness, the microbial community in the JASF group exhibited greater evenness, likely due to a more balanced distribution of dominant species. This observation was further supported by the Pielou_e index, which showed improved species evenness in the JASF group (0.28) compared to the CK group (0.17). Additionally, the Good’s coverage index, approaching 1.000 in both groups, confirmed that the sequencing depth was sufficient to capture the microbial diversity present. These findings suggest that, while fermentation reduced overall species richness, it promoted a more balanced and even microbial community structure, likely driven by changes in substrate availability and microbial competition, indicating a shift in the microbial ecosystem dynamics throughout the fermentation process.

Heatmap analysis (Figure 1B) further highlighted significant differences in microbial community structures between the CK and JASF groups. During the natural fermentation of Jerusalem artichoke tuber juice, the microbial community structure underwent substantial changes. Prior to fermentation, environmental bacteria, such as *Flavobacterium*, *Sphingomonas*, and *Luteimonas*, predominated, participating in the initial degradation of organic matter in the tuber juice. After fermentation, beneficial bacteria like *Lactobacillus* and *Pediococcus* increased significantly, promoting lactic acid production, lowering pH, and thereby inhibiting the growth of harmful bacteria and stabilizing the fermentation environment [46]. *Enterobacter* and *Bacillus* also proliferated during fermentation, and while the former includes some potential pathogens, the latter are mostly beneficial, although certain species like *Bacillus cereus* may cause food poisoning. Therefore, understanding the dynamic changes of beneficial and pathogenic bacteria during fermentation is crucial for ensuring the safety of the fermented product and optimizing the process.

The species diversity and abundance of bacteria at the phylum and genus levels during the spontaneous fermentation of Jerusalem artichoke tubers are displayed in Figure 1C. CK and JASF represent unique bacterial communities specific to each fermentation stage, while CK-JASF represents the bacterial species shared between both groups, highlighting overlapping metabolic activities during fermentation. The microbial community is highly diverse, involving multiple phyla and genera, which is essential for a balanced fermentation process, contributing to the development of flavors, textures, and possibly health benefits of the fermented product. *Proteobacteria* was the dominant phylum in the CK group, indicating its significant role before fermentation, while *Firmicutes* became more prominent in the JASF group, suggesting their increased activity during fermentation. However, *Proteobacteria* in the CK group might include potential pathogens that start to decrease as fermentation progresses, suggesting the need to control such pathogens during natural fermentation. *Firmicutes* are notable in the JASF condition, suggesting their involvement in breaking down complex carbohydrates and producing fermentation end products like lactic acid. Genera such as *Lactobacillus*, *Gluconobacter*, *Lysinibacillus*, and *Pediococcus* in JASF are important for lactic acid production and crucial for lowering pH and inhibiting spoilage organisms [47,48,49]. Differences in microbial composition across CK, JASF, and CK-JASF highlight the impact of environmental factors and fermentation stages on microbial dynamics.

#### 3.1.2. Fungal Diversity

**Figure 2:**
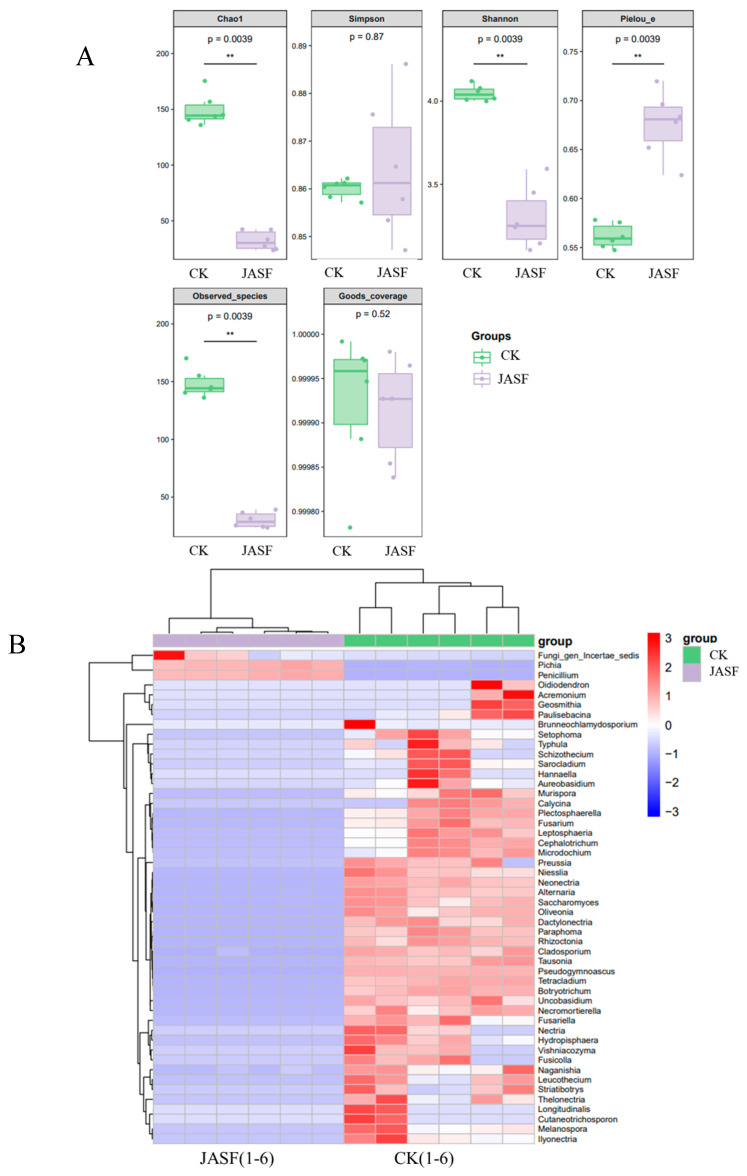

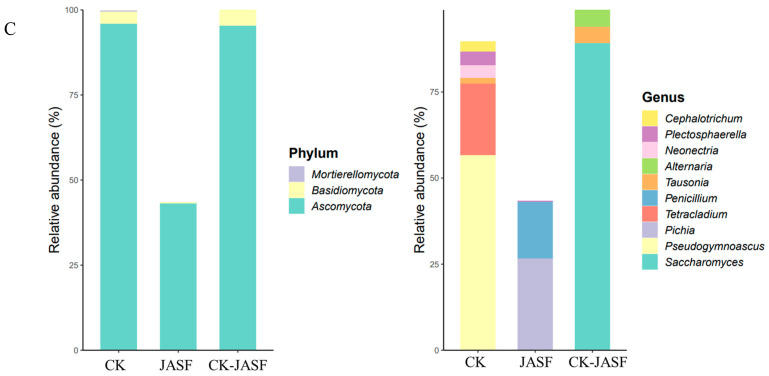
Composition and diversity of fungal communities during spontaneous fermentation. α-diversity index analysis of fungi (**A**). Heatmap of genus-level species composition for co-clustering (**B**). Species diversity and abundance of fungi at the phylum and genus levels (**C**). Statistical significance is indicated by ** (*p* < 0.01).

The α-diversity index analysis of fungi (Figure 2A) shows significant changes in fungal community structure during fermentation. The Chao1 index indicates a significant decrease in fungal richness, from approximately 150 in the CK group to about 32 in the JASF group (*p* = 0.0039). The Shannon index reveals a decrease in diversity and evenness, dropping from about 4.0 in the CK group to 3.3 in the JASF group (*p* = 0.0039), while Pielou’s evenness index increases from 0.56 to 0.68 (*p* = 0.0039). The Simpson index remains similar between groups at around 0.86 (*p* = 0.87), suggesting little change in the dominance of fungal communities. The number of observed species decreases significantly from about 148 to 30 (*p* = 0.0039), consistent with the Chao1 results. The Good’s coverage index, nearly 1.000 for both groups (*p* = 0.52), indicates sufficient sequencing depth. Overall, while fungal richness decreases after fermentation, evenness increases, leading to a more uniform community structure.

Heatmap analysis reveals distinct fungal community structures before (CK group) and after (JASF group) fermentation (Figure 2B). During the natural fermentation of Jerusalem artichoke tuber juice, significant changes in the fungal community structure were observed. Before fermentation (CK group), environmental fungi such as *Geosmithia*, *Paecilomyces*, *Brunneochlamydosporium*, *Alternaria*, and *Cladosporium* dominated, participating in the initial degradation of organic matter. After fermentation, there was a notable increase in *Pichia* and *Penicillium*. *Pichia* proliferated significantly during fermentation, contributing to the stability and flavor development of the product. However, the increase in *Penicillium* raises potential safety concerns due to possible mycotoxin production, which warrants further investigation to ensure product safety [50,51]. Given that certain *Penicillium* species can produce mycotoxins, it is essential to monitor and control their presence during fermentation to ensure the safety of the final product. The fermentation process, characterized by lower pH, organic acid production, and reduced oxygen levels, favored the growth of specific yeasts and molds while inhibiting plant pathogens like *Fusarium* and *Alternaria*. This dynamic shift not only ensures the safety of the fermented product, but also enhances its flavor and quality.

Figure 2C shows the species diversity and abundance of fungi at both the phylum and genus levels across two main groups, CK and JASF, with CK-JASF representing the shared fungal species between both groups. At the phylum level, *Ascomycota* was dominant in all conditions, particularly in CK and CK-JASF, highlighting its critical role during fermentation. This phylum is known for its ability to produce enzymes and secondary metabolites that break down complex carbohydrates and contribute to flavor development [52]. *Basidiomycota* was more abundant in CK but showed a significant reduction in JASF, possibly due to competition from *Ascomycota* species that thrive in acidic fermentation environments. *Mortierellomycota* was present in trace amounts, suggesting a minimal role in the fermentation process. At the genus level, *Saccharomyces* was highly abundant in both CK and CK-JASF, playing a crucial role in fermentation due to its capabilities in ethanol production and flavor enhancement. In contrast, genera such as *Tetracladium* and *Pseudogymnoascus* were more prominent in CK but became less abundant in JASF, implying their involvement in the early stages of fermentation but reduced activity later. *Pichia* and *Penicillium* showed increased abundance in JASF, suggesting their importance in the later stages of fermentation. *Pichia* is recognized for its ability to ferment a wide range of sugars and produce aromatic compounds, while *Penicillium* can break down complex organic materials and potentially produce antimicrobial compounds that inhibit spoilage organisms. Other genera, such as *Cephalotrichum*, *Pleospora*, *Neocallimastix*, *Alternaria*, and *Tausonia*, contributed to the overall fungal diversity. *Cephalotrichum* and *Pleospora* may assist in plant material degradation, while *Neocallimastix* is typically found in the rumens of herbivores and specializes in degrading fibrous plant material; its detection in our samples may suggest contamination or a transient presence, and its role in the fermentation of Jerusalem artichoke juice requires further investigation. *Alternaria* and *Tausonia* can produce various metabolites that may influence the flavor profile of the fermented juice [53,54]. These results highlight the dynamic changes in fungal community composition during fermentation, influenced by pH and substrate availability. The CK-JASF overlap suggests shared metabolic activities, while the differences in CK and JASF reflect distinct microbial processes at different fermentation stages.

### 3.2. Dynamic Changes in Secondary Metabolites during Fermentation

**Figure 3:**
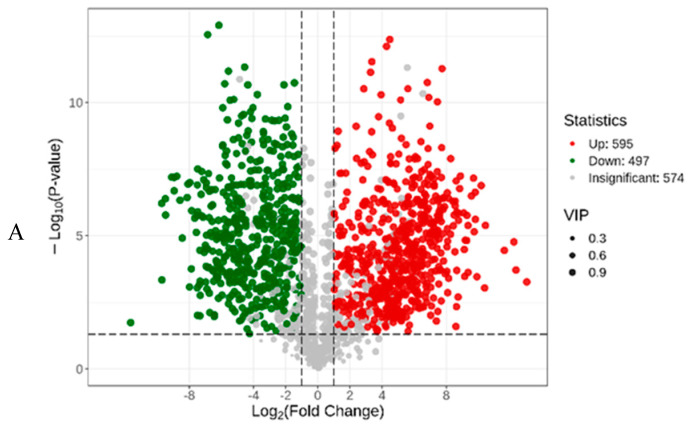

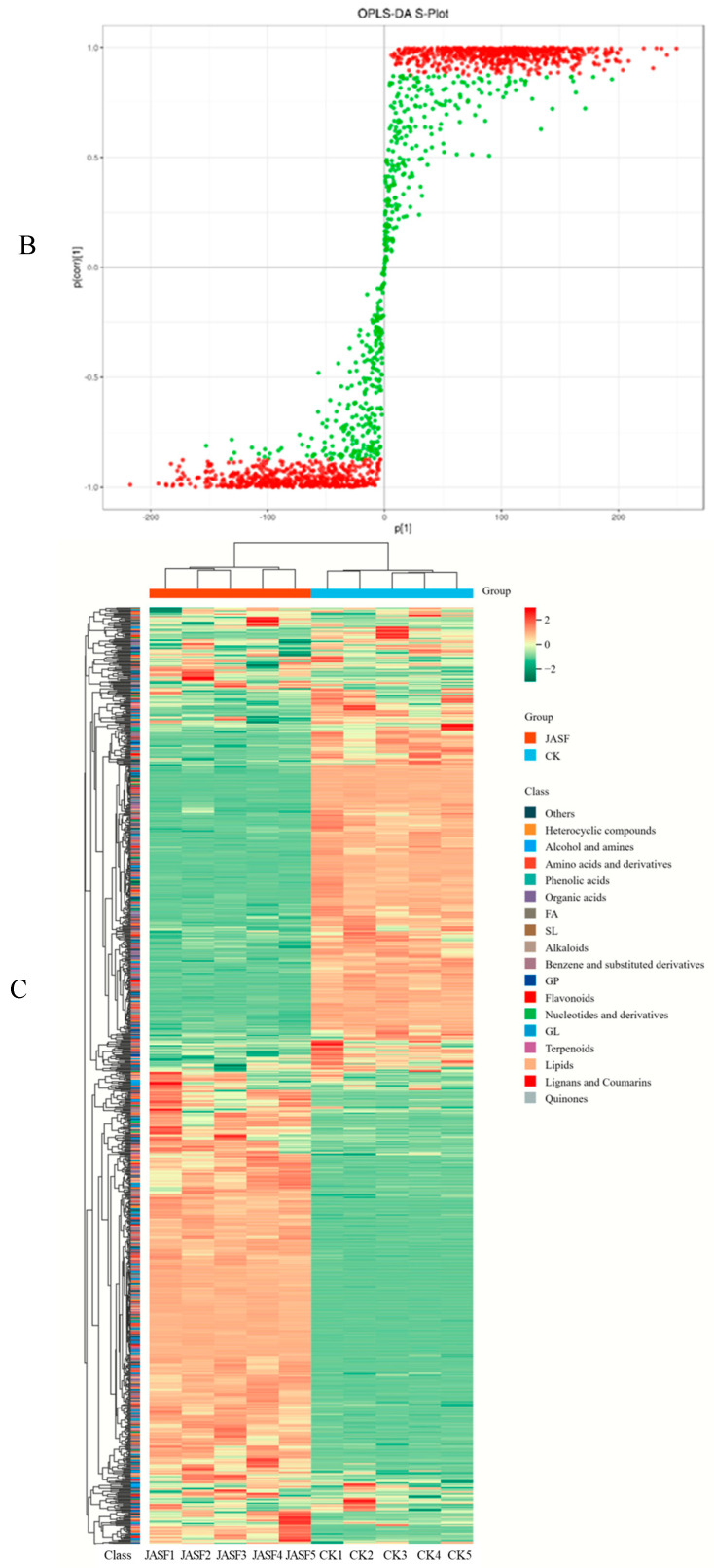

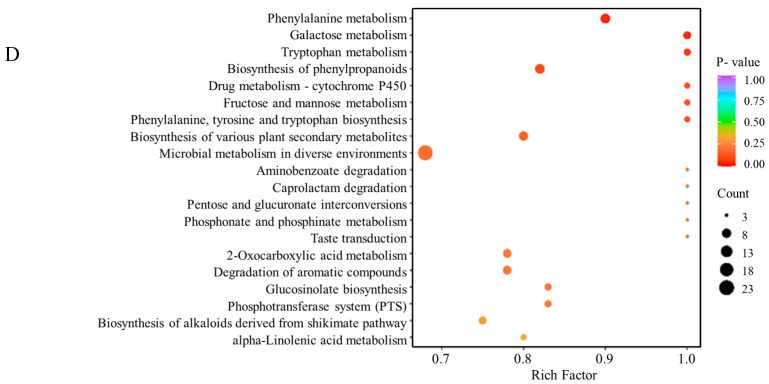
Dynamic changes in secondary metabolites during fermentation. Volcano plot (**A**). The OPLS–DA S−plot (**B**). Heatmap of the differential secondary metabolites (**C**). Enrichment of the differential secondary metabolites (**D**).

The volcano plot (Figure 3A) illustrates the relative abundance differences of metabolites between the CK and JASF groups and the statistical significance of these differences. In this plot, 595 metabolites are significantly upregulated in the experimental group, 497 metabolites are significantly downregulated, and 574 metabolites show no significant difference. The size of the dots indicates the VIP (variable importance in projection) score, with larger dots representing higher VIP scores and suggesting greater importance in distinguishing between the two groups (Table S1). The OPLS–DA S–plot (Figure 3B) provides a visual representation of the metabolites that contribute most significantly to the differences between the CK and JASF groups. Metabolites located closer to the top right and bottom left corners exhibit more significant differences and have VIP values greater than 1, indicating that they are key biomarkers for the fermentation process. These metabolites play a critical role in distinguishing between the CK and JASF groups. Although metabolites with smaller VIP values contribute less to differentiating the two groups, they still affect the overall metabolic profile and may interact with key metabolites.

To further expand on the analysis and discussion of Figure 3C, it is crucial to delve deeper into the implications of the observed shifts in secondary metabolites between the CK and JASF groups. The heatmap clearly indicates that the metabolic landscape of Jerusalem artichoke juice undergoes significant changes during fermentation, with distinct metabolites contributing to different stages of the process [55]. The prominence of amino acids and their derivatives in the CK group suggests that these compounds are vital substrates in the early stages, as they may support the growth of specific microbial communities and facilitate initial microbial proliferation. The subsequent decrease in amino acid levels in the JASF group suggests their consumption during fermentation, either for microbial metabolism or for the biosynthesis of secondary metabolites like alkaloids and terpenoids. Organic acids, such as citric, lactic, and succinic acids, are also essential metabolic intermediates, particularly in maintaining pH balance, which directly affects microbial diversity and enzyme activity. The elevated levels of organic acids in the CK group, followed by their reduction in the JASF group, suggest that these acids are metabolized by microorganisms during fermentation, potentially being converted into alcohols and esters that contribute to flavor development. Additionally, phenolic acids, which are abundant in the CK group, play a protective role against oxidative damage and microbial invasion during the initial stages because of their antioxidant properties. However, as fermentation progresses, phenolic acids may be metabolized or transformed into more complex secondary metabolites, such as flavonoids and alkaloids, which exhibit enhanced bioactivity. A notable increase in alkaloids and terpenoids in the JASF group points to a shift in microbial metabolism under more competitive conditions. These metabolites, often associated with stress responses in both plants and microbes, may help shape the microbial community by inhibiting certain microorganisms and promoting the growth of others. Moreover, heterocyclic compounds and flavonoids, which are more abundant in the JASF group, further support the microbial balance and antioxidant mechanisms. Heterocyclic compounds are involved in microbial signaling pathways, while flavonoids, known for their antioxidant activity, may be synthesized in greater amounts during the later stages of fermentation to protect the matrix from oxidative stress and enhance product stability.

The KEGG pathway enrichment analysis highlights significant metabolic pathway changes between the CK and JASF groups during the natural fermentation of Jerusalem artichoke tuber juice (Figure 3D). Phenylalanine metabolism, galactose metabolism, and tryptophan metabolism pathways are notably enriched, reflecting substantial gene expression changes in these areas. The biosynthesis pathway of phenylpropanoids is also significantly enriched, indicating an increase in secondary metabolites that contribute to flavor and antioxidant properties. The enrichment of drug metabolism via cytochrome P450 and fructose and mannose metabolism pathways underscores active microbial metabolism throughout fermentation. Enhanced degradation pathways for compounds, including aminobenzoate and caprolactam, suggest increased organic compound breakdown and transformation. Additionally, the biosynthesis of various plant secondary metabolites and changes in taste transduction pathways highlight the complexity of biochemical reactions. The metabolism of 2-oxocarboxylic acids and aromatic compounds further emphasizes the dynamic nature of metabolic activities occurring during fermentation. These enriched pathways illustrate the intricate microbial interactions and metabolic processes during fermentation. They enhance the flavor, improve the nutritional value, and contribute to the safety of the fermented product.

### 3.3. Dynamic Changes in Volatile Flavor Compounds

During the spontaneous fermentation of Jerusalem artichoke juice, significant and diverse changes were observed in alcohol compounds, reflecting complex biochemical reactions and microbial metabolic activities during fermentation (Table 1). For example, the content of 3-Undecanol increased from 0.68 μg/L before fermentation to 24.29 μg/L after fermentation, and 1-Octanol increased from 3.79 μg/L to 170.37 μg/L. These significant increases indicate the microbial breakdown and utilization of sugars and other organic substances in Jerusalem artichoke juice during fermentation. Phenylethyl Alcohol showed a particularly significant increase from 113.08 μg/L to 15,675.14 μg/L, with its relative odor activity value (rOAV) also rising markedly. This increase suggests that Phenylethyl Alcohol, associated with floral and fruity aromas, contributes significantly to the post-fermentation product’s fragrance, likely making the juice more appealing to consumers. This change could be attributed to the metabolic activities of yeasts and other microbes, which produce a large amount of secondary metabolites, including alcohols and esters, during sugar breakdown. The total alcohol content increased from 552.14 μg/L to 17,155.1 μg/L post-fermentation, indicating the significant role of fermentation in enhancing the flavor complexity and aroma of Jerusalem artichoke juice. This substantial change reflects vigorous microbial metabolic activity, leading to the generation of numerous new compounds and significant improvements in sensory properties.

During the spontaneous fermentation of Jerusalem artichoke juice, aldehydes showed significant changes, reflecting complex biochemical reactions and microbial metabolic activities. Comparing the data before and after fermentation, 23 aldehyde compounds exhibited notable changes. The content of 10-Undecenal increased from 1.15 μg/L before fermentation to 15.20 μg/L after fermentation, (E,E)-2,4-Nonadienal increased from 10.71 μg/L to 59.63 μg/L, and 2,5-Dimethylbenzaldehyde increased from 21.22 μg/L to 118.21 μg/L. These significant increases indicate the microbial transformation and utilization of precursor substances in Jerusalem artichoke juice during fermentation. In contrast, Benzeneacetaldehyde decreased from 98.40 μg/L to 36.35 μg/L, suggesting a different metabolic pathway or consumption during the fermentation process. The fermentation process led to an overall increase in aldehyde content from 865.39 μg/L to 1172.78 μg/L, highlighting its role in enhancing the flavor complexity and aroma of Jerusalem artichoke juice.

During the spontaneous fermentation of Jerusalem artichoke juice, acid compounds also showed significant changes, demonstrating the impact of microbial metabolic activity and diversity on flavor substances. Analyzing the changes in five major acid compounds provides deeper insights into their contributions to post-fermentation flavor. Hexanoic acid increased from 0.23 μg/L before fermentation to 177.36 μg/L after fermentation, with its rOAV increasing from 0 to 0.06. This significant increase could be due to yeast breaking down fatty acids during fermentation. Hexanoic acid, a common fatty acid with strong fatty and fruity aromas, is often found in fermented beverages. 9-Decenoic acid increased from 0.64 μg/L to 43.76 μg/L. This acid, known for its unique spicy and fruity aromas, may result from the microbial conversion of unsaturated fatty acids. 4-Aminobutanoic acid (γ-aminobutyric acid) increased from 17.57 μg/L to 78.55 μg/L. γ-Aminobutyric acid, an important neurotransmitter with multiple health benefits, is possibly produced through microbial amino acid metabolism, significantly enhancing the product’s health value and flavor. The total acid content increased from 33.27 μg/L to 382.24 μg/L, not only enhancing the sourness and overall flavor, but also potentially improving the product’s antioxidant activity and health benefits.

Ketone compounds showed notable changes during fermentation, highlighting microbial metabolism’s role in flavor development. Analyzing the changes in 24 ketone compounds, we found that Acetophenone increased from 1.28 μg/L before fermentation to 86.86 μg/L after fermentation, with its contribution to flavor becoming more significant. 5-Ethyl-3-Hydroxy-4-Methyl-2(5H)-Furanone increased significantly from 4.13 μg/L to 303.74 μg/L, a highly significant increase, with strong caramel and fruity aromas forming an important aroma substance during fermentation. 2-Methylcyclohexanone increased from 3.56 μg/L to 41.88 μg/L, reflecting its role in the post-fermentation flavor profile. 1-Nonen-3-one increased from 8.76 μg/L to 216.16 μg/L, contributing its unique metallic and sweet aromas, which are commonly found in fruits and vegetables. 4-Undecanone increased from 28.48 μg/L to 170.27 μg/L, further enhancing the flavor profile. In contrast, 3-Methyl-4-Heptanone and 6-Methyl-3,5-Heptadien-2-one decreased from 170.38 μg/L to 72.88 μg/L and from 214.76 μg/L to 167.15 μg/L, respectively. Overall, most ketone compounds significantly increased during fermentation, with the total ketone content rising from 926.95 μg/L to 2195.82 μg/L. This significant increase mainly results from the microbial conversion and metabolic activity of precursor substances in Jerusalem artichoke juice during fermentation. Statistical analysis showed that 20 out of 24 ketone compounds had statistically significant changes (*p* < 0.05), reflecting active microbial metabolism during fermentation and further demonstrating the key role of microbes in flavor substance formation.

Similarly, esters displayed significant alterations, emphasizing the impact of microbial activity on the formation of flavor compounds. Analyzing the changes in 61 ester compounds, various esters showed significant changes before and after fermentation. Ethyl Decanoate increased from 0.06 μg/L before fermentation to 162.31 μg/L after fermentation, contributing significantly to post-fermentation flavor. Ethyl Hexanoate increased from 0.10 μg/L to 160.55 μg/L, enhancing the fruity and sweet aromas of the juice. Octyl Acetate increased from 0.35 μg/L to 61.37 μg/L, adding to the overall fruity aroma. 1-Methylbutyl Butanoate increased from 0.38 μg/L to 69.98 μg/L, and Ethyl Hexadecanoate increased from 0.56 μg/L to 67.52 μg/L, both contributing to the complex aroma profile. Ethyl Benzenepropanoate saw a significant increase from 0.86 μg/L to 2088.29 μg/L, with its floral and sweet aromas enhancing the overall flavor. Ethyl 4-Methylpentanoate increased from 0.59 μg/L to 492.01 μg/L, adding significantly to the fruity aroma. Geranyl Formate increased from 1.16 μg/L to 138.01 μg/L, known for its strong fruity aromas. Pentyl butanoate increased from 1.22 μg/L to 279.13 μg/L, adding banana and fruit aromas to the juice. 1-Isothiocyanato-2-Butene increased from 2.56 μg/L to 311.28 μg/L, and Butyl Butanoate increased from 12.14 μg/L to 233.19 μg/L, both significantly enhancing the aroma. 3-Methylbutyl Butanoate increased from 12.74 μg/L to 1406.55 μg/L, adding to the rich, fruity, and sweet aromas. Ethyl Butanoate increased from 189.73 μg/L to 1056.87 μg/L, contributing to the rich aroma profile. In contrast, some ester compounds showed a decrease during fermentation. (E)-Methyl 3-Hexenoate, Ethyl Tiglate, and Methyl 2-Octynoate saw reductions in their concentrations, indicating changes in the microbial metabolic pathways during fermentation and affecting the overall flavor profile. Most ester compounds significantly increased during fermentation, with the total ester content rising from 1190.37 μg/L to 10,850.27 μg/L. The substantial increase in ester compounds results from microbial esterification processes during fermentation, during which microbes convert alcohols and acids into esters, enhancing the juice’s fruity aroma. Significance analysis showed that most of the 61 ester compounds had statistically significant changes (*p* < 0.05), reflecting active microbial metabolism during fermentation and further demonstrating the key role of microbes in flavor substance formation. High-content ester compounds formed during fermentation, such as hexyl acetate and ethyl decanoate, contributed significantly to the overall aroma and flavor of the fermented product.

During the spontaneous fermentation of Jerusalem artichoke juice, significant changes in various compounds were observed, reflecting complex biochemical reactions and microbial activities. Microbial diversity played a crucial role, with initial stages dominated by *Flavobacterium*, *Sphingomonas*, and *Luteimonas* breaking down complex substances like inulin, leading to flavor development [56]. As fermentation progressed, *Lactobacillus* and *Pediococcus* became dominant, consuming organic acids and synthesizing flavor compounds [57,58]. Fungal shifts from *Geosmithia* and *Alternaria* to *Pichia* and *Penicillium* also contributed significantly, particularly in producing alcohols and esters [59]. Alcohols like phenylethyl alcohol, which showed a dramatic increase, are produced by yeast through the Ehrlich pathway, which converts amino acids to alcohols, while aldehydes like 10-undecenal and 2,5-dimethylbenzaldehyde result from the microbial oxidation of alcohols and amino acids. Acids such as Hexanoic acid and 4-Aminobutanoic acid increase through microbial fermentation, enhancing the juice’s health benefits and antioxidant activity [60]. Ketones, including 5-Ethyl-3-hydroxy-4-methylfuran-2(5H)-one, form via microbial metabolism of fatty acids and amino acids, while esters like Ethyl Decanoate and Ethyl Hexanoate result from microbial esterification, contributing fruity and sweet aromas [61]. The significant role of inulin, a prebiotic, selectively promotes specific microbes, influencing community dynamics and metabolic activities, while reducing sugars are efficiently utilized by *Saccharomyces* and *Pichia*, enhancing the fruity and floral aromas [62]. These findings highlight the critical role of microbial diversity in the development of complex flavor profiles, alongside metabolic pathways and substrate utilization, providing insights for optimizing fermentation processes and improving the quality and flavor of fermented Jerusalem artichoke juice.

## 4. Conclusions

This study revealed the shifts in microbial communities, metabolic transformations, and flavor characteristics during the spontaneous fermentation of Jerusalem artichoke juice. The dominant microbial groups transitioned from environmental bacteria, such as *Flavobacterium* and *Sphingomonas*, to beneficial families like *Lactobacillaceae* and *Acetobacteraceae* during fermentation, with notable post-fermentation increases in *Actinobacteria* and *Bacteroidetes*. Secondary metabolites involved in phenylalanine and tryptophan pathways were enriched and lipid and nucleotide metabolic activities were heightened, indicating increased metabolic activity. The increase in phenylpropanoid and aromatic amino acid metabolites emphasizes their role in enhancing the flavor and potential health benefits of fermented Jerusalem artichoke juice. Flavor analysis showed significant increases in alcohols, esters, acids, and ketones, contributing to the unique aroma and health benefits of fermented Jerusalem artichoke juice.

The main advantage of spontaneous fermentation is its ability to preserve the natural flavor and nutritional components of the raw materials without the need for exogenous strains while producing a rich variety of secondary metabolites. The changes in microbial populations are closely linked to metabolic activities during fermentation, with *Lactobacillaceae* and *Acetobacteraceae* playing pivotal roles in the later stages, significantly influencing the flavor and quality of the juice.

However, spontaneous fermentation is sensitive to environmental factors, leading to challenges in controlling microbial community composition and metabolic pathways, which can result in inconsistencies in product quality. Implementing controlled fermentation with selected starter cultures may help mitigate these issues. Future studies should focus on identifying and utilizing beneficial microbial strains to enhance product consistency and safety. These findings provide a foundation for optimizing fermentation processes by controlling environmental conditions and potentially introducing selected starter cultures to enhance the quality and consistency of fermented Jerusalem artichoke products.

## Figures and Tables

**Table 1:** Changes in volatile flavor compounds during spontaneous fermentation.

Volatile Compounds		RI	CAS	rOAV		Content(μg/L)		*p*-Value
				CK	JASF	CK	JASF	
**Alcohols**								
1	cis-2-Furanmethanol, 5-Ethenyltetrahydro-α,α,5-Trimethyl	1074	5989-33-3	0–1	0–1	0.28 ± 0.02	5.30 ± 0.60	0.0142
2	3-Undecanol	1400	6929-08-4	0–1	>1	0.68 ± 0.02	24.29 ± 0.30	0.0002
3	2,3-Dimethyl-2-Butanol	720	594-60-5	0–1	0–1	1.36 ± 0.05	7.58 ± 0.57	0.0097
4	4,4-Dimethyl-2-Pentanol	812	6144-93-0	0–1	0–1	1.36 ± 0.03	7.58 ± 0.50	0.0058
5	α,α,4-Trimethyl-3-Cyclohexene-1-Methanethiol	1283	71159-90-5	>1	>1	2.83 ± 0.12	15.75 ± 1.09	0.0056
6	2-Ethyl-1-Hexanol	1029	104-76-7	0–1	0–1	3.59 ± 0.24	20.01 ± 1.18	0.0058
7	1-Octanol	1070	111-87-5	0–1	>1	3.79 ± 0.06	170.37 ± 14.04	0.0070
8	1-Undecanol	1371	112-42-5	0–1	0–1	4.13 ± 0.11	7.73 ± 0.40	0.0188
9	2-Mercaptoethanol	723	60-24-2	0–1	0–1	6.70 ± 0.38	37.32 ± 1.46	0.0035
10	1-Decanol	1272	112-30-1	0–1	>1	8.36 ± 0.60	46.58 ± 3.68	0.0119
11	6-Undecanol	1281	23708-56-7	0–1	>1	8.43 ± 0.49	46.95 ± 2.60	0.0030
12	2-Nonanol	1099	628-99-9	0–1	0–1	10.32 ± 0.60	4.20 ± 0.21	0.0045
13	3-Methyl-4-Heptanol	997	1838-73-9	0–1	>1	14.79 ± 0.59	207.21 ± 9.49	0.0027
14	5-Hexen-1-ol	868	821-41-0	0–1	0–1	17.70 ± 0.85	15.85 ± 0.93	0.4025
15	2-Butoxyethanol	905	111-76-2	0–1	0–1	18.04 ± 0.76	100.47 ± 5.98	0.0044
16	2-Heptanol	900	543-49-7	0–1	>1	23.58 ± 1.58	131.35 ± 13.45	0.0189
17	6-Ethenyltetrahydro-2,2,6-Trimethyl-2H-Pyran-3-ol	1173	14049-11-7	0–1	0–1	25.69 ± 0.26	154.34 ± 13.49	0.0112
18	2-Undecanol	1301	1653-30-1	>1	>1	27.97 ± 1.81	155.82 ± 12.67	0.0081
19	trans,cis-2,6-Nonadien-1-ol	1170	28069-72-9	>1	>1	31.48 ± 1.00	126.11 ± 9.14	0.0078
20	Phenylethyl Alcohol	1116	60-12-8	0–1	>1	113.08 ± 10.17	15675.14 ± 834.64	0.0028
21	Hotrienol	1106	20053-88-7	>1	>1	227.97 ± 7.85	195.16 ± 9.53	0.1997
	**Aldehydes**							
22	3-Cyclohexene-1-Carboxaldehyde	958	100-50-5	0–1	0–1	0.39 ± 0.02	2.16 ± 0.01	0.0002
23	(Z)-3-Phenylacrylaldehyde	1219	57194-69-1	0–1	0–1	0.50 ± 0.02	2.79 ± 0.05	0.0002
24	5-Methyl-2-Thiophenecarboxaldehyde	1118	13679-70-4	0–1	>1	0.99 ± 0.05	5.49 ± 0.17	0.0014
25	10-Undecenal	1297	112-45-8	0–1	>1	1.15 ± 0.03	15.20 ± 0.46	0.0011
26	(Z,Z)-3,6-Nonadienal	1100	21944-83-2	>1	>1	1.18 ± 0.13	6.60 ± 0.27	0.0029
27	2-Ethyl-2-Hexenal	999	645-62-5	0–1	0–1	1.97 ± 0.10	2.59 ± 0.09	0.0450
28	Glutaraldehyde	895	111-30-8	>1	>1	4.65 ± 0.34	25.91 ± 0.48	0.0001
29	Piperonal	1334	120-57-0	0–1	0–1	5.07 ± 0.22	3.17 ± 0.22	0.0020
30	(E)-4-Decenal	1198	65405-70-1	0–1	>1	6.00 ± 0.30	39.83 ± 4.79	0.0177
31	3-Methylbenzaldehyde	1070	620-23-5	0–1	0–1	9.27 ± 0.73	51.65 ± 0.22	0.0005
32	Heptanal	901	111-71-7	>1	>1	9.50 ± 0.43	52.90 ± 3.14	0.0067
33	2-Nonenal	1161	2463-53-8	>1	>1	10.29 ± 0.71	57.32 ± 3.67	0.0056
34	(E,E)-2,4-Octadienal	1115	30361-28-5	0–1	0–1	10.71 ± 0.80	59.63 ± 4.57	0.0096
35	4-(1-Methylethenyl)-1-Cyclohexene-1-Carboxaldehyde	1274	2111-75-3	0–1	>1	11.52 ± 0.75	64.19 ± 3.76	0.0063
36	(Z)-6-Nonenal	1104	2277-19-2	>1	>1	14.56 ± 0.33	7.88 ± 0.67	0.0056
37	2,5-Dimethylbenzaldehyde	1154	5779-94-2	0–1	0–1	17.47 ± 0.29	3.89 ± 0.36	0.0002
38	(E,E)-2,4-Nonadienal	1216	5910-87-2	>1	>1	21.22 ± 1.28	118.21 ± 11.23	0.0161
39	Tridecanal	1513	10486-19-8	0–1	0–1	45.38 ± 2.90	53.60 ± 0.37	0.0887
40	(S)-4-(1-Methylethenyl)-1-Cyclohexene-1-Carboxaldehyde	1243	18031-40-8	>1	>1	49.49 ± 2.36	34.55 ± 0.13	0.0240
41	Benzeneacetaldehyde	1046	122-78-1	>1	>1	98.40 ± 2.14	36.35 ± 1.20	0.0026
42	Nonanal	1105	124-19-6	>1	>1	117.08 ± 2.46	90.79 ± 4.02	0.0526
43	(Z)-2-Decenal	1252	2497-25-8	>1	>1	170.39 ± 13.31	128.60 ± 2.42	0.0627
44	4-(1,1-Dimethylethyl)benzenepropanal	1521	18127-01-0	>1	>1	258.20 ± 14.06	309.48 ± 23.63	0.2868
	**Acids**							
45	Hexanoic Acid	987	142-62-1	0–1	0–1	0.23 ± 0.01	177.36 ± 8.39	0.0022
46	9-Decenoic Acid	1360	14436-32-9	0–1	0–1	0.64 ± 0.06	43.76 ± 4.64	0.0111
47	4-Methyloctanoic Acid	1232	54947-74-9	0–1	0–1	2.35 ± 0.03	13.06 ± 0.81	0.0054
48	(E)-2-Hexenoic Acid	1045	13419-69-7	0–1	0–1	12.48 ± 0.95	69.51 ± 1.08	0.0001
49	4-Aminobutanoic Acid	1190	56-12-2	0–1	0–1	17.57 ± 1.88	78.55 ± 2.82	0.0010
	**Ketones**							
50	2-Undecanone	1295	112-12-9	0–1	>1	0.58 ± 0.03	10.70 ± 0.42	0.0020
51	Isophorone	1123	78-59-1	0–1	0–1	1.24 ± 0.10	6.92 ± 0.39	0.0029
52	1-(4-Methylphenyl)ethanone	1183	122-00-9	0–1	0–1	1.25 ± 0.05	6.00 ± 0.09	0.0001
53	Acetophenone	1068	98-86-2	0–1	>1	1.28 ± 0.13	86.86 ± 10.04	0.0131
54	2-Dodecanone	1395	6175-49-1	0–1	0–1	1.47 ± 0.10	4.93 ± 0.23	0.0022
55	(E,E)-3,5-Octadien-2-one	1073	30086-02-3	>1	>1	2.31 ± 0.04	12.87 ± 1.30	0.0150
56	(E)-5,9-Undecadien-2-one, 6,10-Dimethyl	1453	3796-70-1	0–1	0–1	3.35 ± 0.11	2.62 ± 0.06	0.0502
57	2-Octanone	991	111-13-7	0–1	0–1	3.50 ± 0.28	19.48 ± 1.15	0.0030
58	2-Methylcyclohexanone	953	583-60-8	0–1	0–1	3.56 ± 0.09	41.88 ± 2.23	0.0036
59	5-Ethyl-3-Hydroxy-4-Methyl-2(5H)-Furanone	1195	698-10-2	>1	>1	4.13 ± 0.33	303.74 ± 23.36	0.0060
60	1-(4,5-Dihydro-2-Thiazolyl)ethanone	1106	29926-41-8	>1	>1	4.64 ± 0.06	25.86 ± 2.12	0.0096
61	3-Butylisobenzofuran-1(3H)-one	1656	6066-49-5	0–1	0–1	5.33 ± 0.39	3.17 ± 0.21	0.0427
62	1-Nonen-3-one	1076	24415-26-7	>1	>1	8.76 ± 0.79	216.16 ± 8.62	0.0015
63	3-Octen-2-one	1016	1669-44-9	>1	>1	11.08 ± 0.28	61.71 ± 4.26	0.0079
64	3-Decanone	1187	928-80-3	0–1	>1	13.10 ± 0.32	72.95 ± 2.82	0.0022
65	4-(2,6,6-Trimethylcyclohexa-1,3-Dienyl)but-3-en-2-one	1485	1203-08-3	>1	>1	14.06 ± 1.51	6.96 ± 0.06	0.0403
66	1-(2,6,6-Trimethyl-1,3-Cyclohexadien-1-yl)-2-Buten-1-one	1362	23696-85-7	>1	>1	17.67 ± 0.33	17.37 ± 1.11	0.8407
67	1-(2-Thienyl)ethanone	1092	88-15-3	>1	>1	17.95 ± 0.88	43.99 ± 0.52	0.0010
68	4-Undecanone	1208	14476-37-0	0–1	>1	28.48 ± 2.43	170.27 ± 9.34	0.0024
69	2-Sec-Butylcyclohexanone	1220	14765-30-1	0–1	>1	75.95 ± 3.34	423.10 ± 13.08	0.0022
70	2-Hydroxy-3,4-Dimethyl-2-Cyclopenten-1-one	1075	21835-00-7	>1	>1	141.02 ± 4.04	136.87 ± 10.86	0.8055
71	3-Methyl-4-Heptanone	928	15726-15-5	>1	>1	170.38 ± 17.84	72.88 ± 1.21	0.0287
72	3,4-Dimethyl-1,2-Cyclopentadione	1109	13494-06-9	>1	>1	181.10 ± 9.76	281.39 ± 4.42	0.0192
73	6-Methyl-3,5-Heptadien-2-one	1107	1604-28-0	>1	>1	214.76 ± 8.41	167.15 ± 14.82	0.1383
	**Esters**							
74	Ethyl Decanoate	1396	110-38-3	0–1	>1	0.06 ± 0.00	162.31 ± 13.54	0.0069
75	Ethyl Hexanoate	999	123-66-0	0–1	>1	0.10 ± 0.01	160.55 ± 3.85	0.0006
76	Octyl Acetate	1210	112-14-1	0–1	0–1	0.35 ± 0.05	61.37 ± 5.29	0.0074
77	1-Methylbutyl Butanoate	970	60415-61-4	0–1	>1	0.38 ± 0.02	69.98 ± 5.37	0.0059
78	Ethyl Hexadecanoate	1993	628-97-7	0–1	0–1	0.56 ± 0.01	67.52 ± 4.04	0.0036
79	Ethyl 4-Methylpentanoate	969	25415-67-2	0–1	>1	0.59 ± 0.05	492.01 ± 27.89	0.0032
80	1-Methylpropyl 2-Methylbutanoate	971	869-08-9	0–1	0–1	0.78 ± 0.05	4.32 ± 0.12	0.0021
81	Ethyl Benzenepropanoate	1353	2021-28-5	0–1	>1	0.86 ± 0.05	2088.29 ± 67.40	0.0010
82	4-tert-Butylcyclohexyl Acetate	1368	32210-23-4	0–1	0–1	0.90 ± 0.08	23.44 ± 1.30	0.0037
83	Ethyl 9-Decenoate	1388	67233-91-4	0–1	0–1	1.00 ± 0.00	34.92 ± 3.26	0.0091
84	2-Ethylhexyl Acrylate	1220	103-11-7	0–1	0–1	1.14 ± 0.08	14.04 ± 0.53	0.0019
85	Geranyl Formate	1301	105-86-2	0–1	0–1	1.16 ± 0.09	138.01 ± 2.15	0.0002
86	Pentyl Butanoate	1077	540-18-1	0–1	0–1	1.22 ± 0.07	279.13 ± 7.96	0.0008
87	Hexyl Acetate	1013	142-92-7	0–1	0–1	1.24 ± 0.06	6.90 ± 0.09	0.0002
88	Pentyl 2-Methylbutanoate	1142	68039-26-9	0–1	>1	1.25 ± 0.06	24.67 ± 1.29	0.0028
89	Methyl 4-Methoxybenzoate	1373	121-98-2	0–1	0–1	1.58 ± 0.08	3.45 ± 0.11	0.0096
90	trans-3-Methyl-4-Octanolide	1288	39638-67-0	0–1	0–1	1.86 ± 0.07	0.41 ± 0.02	0.0034
91	Methyl Anthranilate	1349	134-20-3	0–1	>1	1.92 ± 0.12	100.47 ± 0.93	0.0001
92	Butyl 2-Hydroxybenzoate	1436	2052-14-4	0–1	0–1	1.93 ± 0.11	2.13 ± 0.13	0.4387
93	Hexyl 2-Methylbutanoate	1236	10032-15-2	0–1	0–1	2.00 ± 0.11	1.62 ± 0.04	0.0323
94	1,2-Ethanediol, Diacetate	991	111-55-7	0–1	0–1	2.03 ± 0.14	11.28 ± 1.06	0.0145
95	2-Ethylhexyl Methacrylate	1296	688-84-6	0–1	0–1	2.15 ± 0.17	28.94 ± 1.30	0.0029
96	1-Isothiocyanato-2-Butene	1070	2253-93-2	0–1	0–1	2.56 ± 0.03	311.28 ± 18.03	0.0034
97	Phenyl Acetate	1062	122-79-2	0–1	0–1	2.61 ± 0.12	10.34 ± 0.86	0.0091
98	2-Ethylhexyl Acetate	1185	103-09-3	0–1	0–1	2.94 ± 0.11	16.37 ± 0.50	0.0009
99	1-Ethylpropyl Acetate	793	620-11-1	0–1	>1	3.10 ± 0.34	194.85 ± 4.87	0.0006
100	δ-Dodecalactone	1720	713-95-1	0–1	0–1	3.45 ± 0.17	4.04 ± 0.04	0.0464
101	Methyl Heptanoate	1024	106-73-0	0–1	>1	3.56 ± 0.12	19.85 ± 0.98	0.0044
102	Ethyl Dodecanoate	1595	106-33-2	0–1	0–1	4.27 ± 0.20	23.78 ± 1.75	0.0095
103	3-Phenylpropyl Acetate	1373	122-72-5	0–1	0–1	4.28 ± 0.07	6.10 ± 0.56	0.0701
104	3-Methylphenylmethyl Butanoate	1396	103-38-8	0–1	>1	5.49 ± 0.50	30.60 ± 1.68	0.0027
105	cis-2-Methyl-5-(1-Methylethenyl)-2-Cyclohexen-1-ol Acetate	1362	1205-42-1	0–1	>1	5.63 ± 0.24	31.37 ± 5.71	0.0463
106	Butyl Hexanoate	1189	626-82-4	0–1	0–1	6.39 ± 0.30	32.25 ± 0.62	0.0004
107	Pentyl Acetate	916	628-63-7	0–1	0–1	6.69 ± 0.32	37.27 ± 2.74	0.0072
108	4-Methylphenyl Acetate	1171	140-39-6	0–1	>1	8.00 ± 0.33	36.87 ± 1.79	0.0051
109	3-Methylbutyl Butanoate	1046	109-19-3	0–1	0–1	8.41 ± 0.45	46.84 ± 1.05	0.0015
110	Ethyl Nonanoate	1295	123-29-5	0–1	>1	9.00 ± 0.58	50.16 ± 2.23	0.0038
111	Methyl Thiocyanate	702	556-64-9	0–1	>1	9.51 ± 0.92	52.95 ± 1.39	0.0026
112	Ethyl 3-Methylpentanoate	960	5870-68-8	>1	>1	9.52 ± 0.93	295.96 ± 2.07	0.0000
113	Tetrahydro-6-Pentyl-2H-Pyran-2-one	1502	705-86-2	0–1	0–1	10.89 ± 0.36	43.16 ± 2.44	0.0069
114	Methyl Decanoate	1326	110-42-9	>1	>1	11.03 ± 0.30	17.51 ± 0.57	0.0172
115	2-Methylbutyl 2-Methylbutanoate	1105	2445-78-5	0–1	>1	11.63 ± 1.03	148.48 ± 6.08	0.0023
116	2-Phenylethyl 3-Methylbutanoate	1491	140-26-1	>1	>1	11.65 ± 1.16	64.87 ± 3.29	0.0043
117	Butyl Butanoate	996	109-21-7	0–1	>1	12.14 ± 0.26	233.19 ± 15.59	0.0049
118	Ethyl Pentanoate	902	539-82-2	0–1	>1	12.16 ± 0.18	67.76 ± 3.27	0.0033
119	Ethyl Benzeneacetate	1247	101-97-3	0–1	0–1	12.52 ± 1.44	71.91 ± 3.06	0.0016
120	Dihydro-5-Propyl-2(3H)-Furanone	1156	105-21-5	0–1	0–1	12.71 ± 0.46	70.78 ± 3.50	0.0028
121	3-Methylbutyl Butanoate	1056	106-27-4	0–1	>1	12.74 ± 0.12	1406.55 ± 18.28	0.0002
122	n-Amyl Isovalerate	1110	25415-62-7	0–1	0–1	18.84 ± 0.91	473.98 ± 30.74	0.0044
123	Isothiocyanatoethane	796	542-85-8	0–1	0–1	20.83 ± 0.96	205.69 ± 30.54	0.0247
124	Propyl Propanoate	810	106-36-5	0–1	>1	22.72 ± 0.54	126.54 ± 7.70	0.0051
125	3-Methyl-1-Butanol Acetate	878	123-92-2	>1	>1	24.93 ± 0.67	471.94 ± 35.83	0.0066
126	Propyl Butanoate	899	105-66-8	>1	>1	30.20 ± 0.85	168.23 ± 4.58	0.0010
127	(E)-Methyl 3-Hexenoate	920	13894-61-6	0–1	0–1	34.56 ± 1.43	7.70 ± 0.67	0.0009
128	n-Butyl Tiglate	1134	7785-66-2	>1	>1	51.65 ± 2.22	287.71 ± 2.48	0.0001
129	Isopentyl Hexanoate	1250	2198-61-0	0–1	0–1	59.91 ± 1.01	39.01 ± 0.71	0.0023
130	Butyl Acetate	815	123-86-4	>1	>1	99.48 ± 5.70	554.18 ± 17.65	0.0017
131	2-Butoxyethyl Acetate	1090	112-07-2	0–1	0–1	102.40 ± 4.71	199.72 ± 6.11	0.0118
132	Ethyl Tiglate	939	5837-78-5	>1	>1	150.65 ± 11.45	66.08 ± 2.19	0.0223
133	Methyl 2-Octynoate	1202	111-12-6	>1	>1	156.55 ± 12.81	91.76 ± 6.52	0.0758
134	Ethyl Butanoate	802	105-54-4	>1	>1	189.73 ± 15.80	1056.87 ± 79.69	0.0109

## Data Availability

Data are contained within the article or Supplementary Material.

## Supplementary Materials

The following supporting information can be downloaded at: https://www.mdpi.com/article/10.3390/plants13192782/s1, Table S1. Differential Metabolite Screening Results.

## References

[1] Chyc M., Ogonowski J. (2015). Jerusalem artichoke as a prospective raw material for industry. Przem. Chem..

[2] Qin Y.-Q., Wang L.-Y., Yang X.-Y., Xu Y.-J., Fan G., Fan Y.-G., Ren J.-N., An Q., Li X. (2023). Inulin: Properties and health benefits. Food Funct..

[3] Riva A., Rasoulimehrabani H., Cruz-Rubio J.M., Schnorr S.L., von Baeckmann C., Inan D., Nikolov G., Herbold C.W., Hausmann B., Pjevac P. (2023). Identification of inulin-responsive bacteria in the gut microbiota via multi-modal activity-based sorting. Nat. Commun..

[4] Tawfick M.M., Xie H., Zhao C., Shao P., Farag M.A. (2022). Inulin fructans in diet: Role in gut homeostasis, immunity, health outcomes and potential therapeutics. Int. J. Biol. Macromol..

[5] Mitchell C.M., Davy B.M., Ponder M.A., McMillan R.P., Hughes M.D., Hulver M.W., Neilson A.P., Davy K.P. (2021). Prebiotic Inulin Supplementation and Peripheral Insulin Sensitivity in adults at Elevated Risk for Type 2 Diabetes: A Pilot Randomized Controlled Trial. Nutrients.

[6] Guimaraes J.B., Rodrigues V.F., Pereira I.S., Manso G.M.d.C., Elias-Oliveira J., Leite J.A., Waldetario M.C.G.M., de Oliveira S., Gomes A.B.d.S.P., Faria A.M.C. (2024). Inulin prebiotic ameliorates type 1 diabetes dictating regulatory T cell homing via CCR4 to pancreatic islets and butyrogenic gut microbiota in murine model. J. Leukoc. Biol..

[7] Jia S., Li J., Yu B., Li M., Cui B. (2023). Improvement of myocardial injury and gut microbiota disturbance in type 2 diabetic mice by inulin with various degrees of polymerization. Food Biosci..

[8] Tang Z., Shao T., Gao L., Yuan P., Ren Z., Tian L., Liu W., Liu C., Xu X., Zhou X. (2023). Structural elucidation and hypoglycemic effect of an inulin-type fructan extracted from *Stevia rebaudiana* roots. Food Funct..

[9] Bakirhan H., Karabudak E. (2023). Effects of inulin on calcium metabolism and bone health. Int. J. Vitam. Nutr. Res..

[10] Gomez-Betancur A.M., Carmona-Tamayo R., Martinez-Alyarez O.L., Casanova-Yepes H., Torres-Oquendo J.D. (2022). Effect of fat substitution using long-chain inulin and fortification with microencapsulated calcium in the rheological and sensory properties of yogurt mousse. J. Food Process Eng..

[11] Sulejmani E., Boran O.S., Huppertz T., Hayaloglu A.A. (2021). Rheology, microstructure and sensory properties of low-fat milk jam: Influence of inulin type, sucrose content, sodium bicarbonate and calcium chloride. Int. Dairy J..

[12] Kowalczyk M., Znamirowska-Piotrowska A., Buniowska-Olejnik M., Zagula G., Pawlos M. (2023). Bioavailability of Macroelements from Synbiotic Sheep’s Milk Ice Cream. Nutrients.

[13] Omori K., Miyakawa H., Watanabe A., Nakayama Y., Lyu Y., Ichikawa N., Sasaki H., Shibata S. (2021). The Combined Effects of Magnesium Oxide and Inulin on Intestinal Microbiota and Cecal Short-Chain Fatty Acids. Nutrients.

[14] Takeuchi J., Nagashima T. (2011). Preparation of dried chips from Jerusalem artichoke (*Helianthus tuberosus*) tubers and analysis of their functional properties. Food Chem..

[15] Showkat M.M., Falck-Ytter A.B., Straetkvern K.O. (2019). Phenolic Acids in Jerusalem Artichoke (*Helianthus tuberosus* L.): Plant Organ Dependent Antioxidant Activity and Optimized Extraction from Leaves. Molecules.

[16] Dias N.S., Ferreira J.F.S., Liu X., Suarez D.L. (2016). Jerusalem artichoke (*Helianthus tuberosus* L.) maintains high inulin, tuber yield, and antioxidant capacity under moderately-saline irrigation waters. Ind. Crops Prod..

[17] Mu Y., Gao W., Lv S., Li F., Lu Y., Zhao C. (2021). The antioxidant capacity and antioxidant system of Jerusalem artichoke (*Helianthus tuberosus* L.) tubers in relation to inulin during storage at different low temperatures. Ind. Crops Prod..

[18] Kolniak-Ostek J., Kita A., Peksa A., Wawrzyniak A., Hamulka J., Jeznach M., Danilcenko H., Jariene E. (2017). Analysis of the content of bioactive compounds in selected flours and enriched extruded corn products. J. Food Compos. Anal..

[19] Zalan Z., Hudacek J., Toth-Markus M., Husova E., Solichova K., Hegyi F., Plockova M., Chumchalova J., Halasz A. (2011). Sensorically and antimicrobially active metabolite production of *Lactobacillus* strains on Jerusalem artichoke juice. J. Sci. Food Agric..

[20] Celik I., Isik F., Gursoy O., Yilmaz Y. (2013). Use of Jerusalem artichoke (*Helianthus Tuberosus*) tubers as a natural source of inulin in cakes. J. Food Process. Preserv..

[21] Radovanovic A., Stojceska V., Plunkett A., Jankovic S., Milovanovic D., Cupara S. (2015). The use of dry Jerusalem artichoke as a functional nutrient in developing extruded food with low glycaemic index. Food Chem..

[22] Diaz A., Bomben R., Dini C., Vina S.Z., Garcia M.A., Ponzi M., Comelli N. (2019). Jerusalem artichoke tuber flour as a wheat flour substitute for biscuit elaboration. Lwt-Food Sci. Technol..

[23] Diaz A., Garcia M.A., Dini C. (2022). Jerusalem artichoke flour as food ingredient and as source of fructooligosaccharides and inulin. J. Food Compos. Anal..

[24] Dimitrovski D., Velickova E., Dimitrovska M., Langerholc T., Winkelhausen E. (2016). Synbiotic functional drink from Jerusalem artichoke juice fermented by probiotic *Lactobacillus plantarum* PCS26. J. Food Sci. Technol..

[25] Sooresh M.M., Willing B.P., Bourrie B.C.T. (2023). Opportunities and challenges of understanding community assembly in spontaneous food fermentation. Foods.

[26] Gao Q., Song Y., Liang Y., Li Y., Chang Y., Ma R., Cao X., Wang S. (2022). Dynamics of physicochemical properties, functional compounds and antioxidant capacity during spontaneous fermentation of *Lycium ruthenicum* Murr. (Qinghai-Tibet Plateau) natural vinegar. Foods.

[27] Zheng Z., Zhou Q., Chen Q., Gao J., Wu Y., Yang F., Zhong K., Gao H. (2023). Improvement of physicochemical characteristics, flavor profiles and functional properties in Chinese radishes via spontaneous fermentation after drying. J. Food Sci..

[28] Lu Y., Sun F., Wang W., Liu Y., Wang J., Sun J., Mu J., Gao Z. (2020). Effects of spontaneous fermentation on the microorganisms diversity and volatile compounds during ‘Marselan’ from grape to wine. Lwt-Food Sci. Technol..

[29] Kumar S., Chhabra V., Shenoy S., Daksh R., Ravichandiran V., Swamy R.S., Kumar N. (2024). Role of flavonoids in modulation of mitochondria dynamics during oxidative stress. Mini-Rev. Med. Chem..

[30] Li W., Zhang X., Wang S., Gao X., Zhang X. (2024). Research progress on extraction and detection technologies of flavonoid compounds in Foods. Foods.

[31] Li J., Lu X., Zou X., Ye B.C. (2024). Recent advances in microbial metabolic engineering for production of natural phenolic acids. J. Agric. Food Chem..

[32] Gao Y., Ma S., Dai M., Feng X.Y. (2018). Progress in research on the biosynthesis pathway and metabolic regulation of phenolic acids. Food Sci. China.

[33] Yang X., Lan W., Sun X. (2023). Antibacterial and antioxidant properties of phenolic acid grafted chitosan and its application in food preservation: A review. Food Chem..

[34] Saini A., Seni K., Chawla P.A., Chawla V., Ganti S.S. (2024). An insight into recent updates on analytical techniques for bioactive alkaloids. Phytochem. Anal..

[35] Zhu R., Zhang J., Lyu Y., Chen Y., Han S., Wang H. (2024). Efficacy of substances containing 3 types of active ingredientssaponins, flavones, and alkaloids in regulation of cytokines in autoimmune diseases a systematic review and Meta-analysis based on animal studies. J. Tradit. Chin. Med..

[36] Kusuma G.D., Paseephol T., Sherkat F. (2009). Prebiotic and rheological effects of Jerusalem artichoke inulin in low-fat yogurt. Aust. J. Dairy Technol..

[37] Yi H., Zhang L., Hua C., Sun K., Zhang L. (2010). Extraction and enzymatic hydrolysis of inulin from Jerusalem artichoke and their effects on textural and sensorial characteristics of yogurt. Food Bioprocess Tech.

[38] Canbulat Z., Ozcan T. (2015). Effects of short-chain and long-chain inulin on the quality of probiotic yogurt containing *Lactobacillus Rhamnosus*. J. Food Process. Preserv..

[39] Guven M., Yasar K., Karaca O.B., Hayaloglu A.A. (2005). The effect of inulin as a fat replacer on the quality of set-type low-fat yogurt manufacture. Int. J. Dairy Technol..

[40] Ehsani J., Mohsenzadeh M., Khomeiri M., Ghasemnezhad A. (2018). Chemical characteristics, and effect of inulin extracted from artichoke (*Cynara scolymus* L.) root on biochemical properties of synbiotic yogurt at the end of fermentation. Iran. J. Chem. Chem. Eng. -Int. Engl. Ed..

[41] Balcazar-Zumaeta C.R., Castro-Alayo E.M., Cayo-Colca I.S., Idrogo-Vasquez G., Munoz-Astecker L.D. (2023). Metabolomics during the spontaneous fermentation in cocoa (*Theobroma cacao* L.): An exploraty review. Food Res. Int..

[42] Luis Navarrete-Bolanos J. (2012). Improving traditional fermented beverages: How to evolve from spontaneous to directed fermentation. Eng. Life Sci..

[43] Yao Z., Zhu Y., Wu Q., Xu Y. (2024). Challenges and perspectives of quantitative microbiome profiling in food fermentations. Crit. Rev. Food Sci. Nutr..

[44] Shi X., Liu Y., Ma Q., Wang J., Luo J., Suo R., Sun J. (2022). Effects of low temperature on the dynamics of volatile compounds and their correlation with the microbial succession during the fermentation of Longyan wine. LWT.

[45] Callahan B.J., Wong J., Heiner C., Oh S., Theriot C.M., Gulati A.S., McGill S.K., Dougherty M.K. (2019). High-throughput amplicon sequencing of the full-length 16S rRNA gene with single-nucleotide resolution. Nucleic Acids Res..

[46] Peter S.B., Qiao Z., Godspower H.N., Ajeje S.B., Xu M., Zhang X., Yang T., Rao Z. (2022). Biotechnological innovations and therapeutic application of Pediococcus and lactic acid bacteria: The next-generation microorganism. Front. Bioeng. Biotechnol..

[47] Medina E., Pérez-Díaz I.M., Breidt F., Hayes J., Franco W., Butz N., Azcarate-Peril M.A. (2016). Bacterial ecology of fermented cucumber rising pH spoilage as determined by nonculture-based methods. J. Food Sci..

[48] Sengun I.Y., Doyle M.P. (2017). Microbiology of fermented foods. Acetic Acid Bacteria.

[49] Paramithiotis S. (2021). Microorganisms associated with food fermentation. Bioactive Compounds in Fermented Foods.

[50] Zhao H., Li Y., Liu L., Zheng M., Feng Z., Hu K., Tao Y. (2022). Effects of inoculation timing and mixed fermentation with *Pichia* fermentans on *Oenococcus* oeni viability, fermentation duration and aroma production during wine malolactic fermentation. Food Res. Int..

[51] Li Y., Nguyen T.T.H., Jin J., Lim J., Lee J., Piao M., Mok I.-K., Kim D. (2021). Brewing of glucuronic acid-enriched apple cider with enhanced antioxidant activities through the co-fermentation of yeast (*Saccharomyces cerevisiae* and *Pichia kudriavzevii*) and bacteria (*Lactobacillus plantarum*). Food Sci Biotechnol.

[52] Debeljak P., Baltar F. (2023). Fungal diversity and community composition across ecosystems. J. Fungi.

[53] Li K., Tang J., Zhang Z., Wu Z., Zhong A., Li Z., Wang Y. (2022). Correlation between flavor compounds and microorganisms of Chaling natural fermented red sufu. Lwt.

[54] Wang X., Schlatter D.C., Glawe D.A., Edwards C.G., Weller D.M., Paulitz T.C., Abatzoglou J.T., Okubara P.A. (2021). Native yeast and non-yeast fungal communities of Cabernet Sauvignon berries from two Washington State vineyards, and persistence in spontaneous fermentation. Int. J. Food Microbiol..

[55] Cao H., Chen B.H., Inbaraj B.S., Chen L., Alvarez-Rivera G., Cifuentes A., Zhang N., Yang D.J., Simal-Gandara J., Wang M. (2020). Preventive potential and mechanism of dietary polyphenols on the formation of heterocyclic aromatic amines. Food Front..

[56] Beygmoradi A., Homaei A. (2017). Marine microbes as a valuable resource for brand new industrial biocatalysts. Biocatal. Agr. Biotech..

[57] Hu Y., Zhang L., Wen R., Chen Q., Kong B. (2022). Role of lactic acid bacteria in flavor development in traditional Chinese fermented foods: A review. Crit. Rev. Food Sci. Nutr..

[58] Song G., He Z., Wang X., Zhao M., Cao X., Lin X., Ji C., Zhang S., Liang H. (2021). Improving the quality of Suancai by inoculating with *Lactobacillus plantarum* and *Pediococcus pentosaceus*. Food Res. Int..

[59] Mohamed H., Hassane A., Atta O., Song Y. (2021). Deep learning strategies for active secondary metabolites biosynthesis from fungi: Harnessing artificial manipulation and application. Biocatal Agr Biotech.

[60] Grewal J. (2020). Gamma-aminobutyric acid (GABA): A versatile bioactive compound. Eur. J. Mol. Clin. Med..

[61] Tang M., Wang Z., Luo J., Zhu T., Song F., Chen H. (2024). Preparation, chemical profiles, antioxidative activities, and angiotensin-converting enzyme 2 inhibitory effect of date fruit vinegar. J. Food Sci..

[62] Keșa A.L., Pop C.R., Mudura E., Salanță L.C., Pasqualone A., Dărab C., Burja-Udrea C., Zhao H., Coldea T.E. (2021). Strategies to improve the potential functionality of fruit-based fermented beverages. Plants.

